# Supplementary material for: COZOID: contact zone identifier for visual analysis of protein-protein interactions
Source: BMC Bioinformatics. 2018 Apr 6;19:125. doi: 10.1186/s12859-018-2113-6 (PMC5889581; doi:10.1186/s12859-018-2113-6)
Supplement: Supplementary file 2 — Software build. Executable binary file of the software tool. (ZIP 118,784 kb) [file 12859_2018_2113_MOESM2_ESM.zip › cozoid/java/docs/beansbinding-1.2.1-doc.zip!/org/jdesktop/beansbinding/Binding.SyncFailure.html]

Binding.SyncFailure (Beans Binding)


---


|  |  |  |
| --- | --- | --- |
| **Overview** \| **Package** \| **Class** \| **Use** \| **Tree** \| **Deprecated** \| **Index** \| **Help** | |  |
| **PREV CLASS**   **NEXT CLASS** | **FRAMES**    **NO FRAMES**     **All Classes** |  |
| SUMMARY: NESTED \| FIELD \| CONSTR \| METHOD | DETAIL: FIELD \| CONSTR \| METHOD |  |


---


## org.jdesktop.beansbinding Class Binding.SyncFailure

```
java.lang.Object
  org.jdesktop.beansbinding.Binding.SyncFailure
```

**Enclosing class:**: Binding<SS,SV,TS,TV>

---

``` public static final class Binding.SyncFailure extends java.lang.Object ```

`SyncFailure` represents a failure to sync (`save` or `refresh`) a
`Binding`.

---

| **Method Summary** | |
| --- | --- |
| `java.lang.RuntimeException` | `getConversionException()`             Returns the exception that occurred during conversion if this failure represents a conversion failure. |
| `Binding.SyncFailureType` | `getType()`             Returns the type of failure. |
| `Validator.Result` | `getValidationResult()`             Returns the result that was returned from the `Binding's` validator if this failure represents a validation failure. |
| `java.lang.String` | `toString()`             Returns a string representation of the `SyncFailure`. |

**Methods inherited from class java.lang.Object**

`clone, equals, finalize, getClass, hashCode, notify, notifyAll, wait, wait, wait`

**Method Detail**

### getType

```
public Binding.SyncFailureType getType()
```

:   Returns the type of failure.

    :   **Returns:**: the type of failure

---


### getConversionException

```
public java.lang.RuntimeException getConversionException()
```

:   Returns the exception that occurred during conversion if
    this failure represents a conversion failure. Throws
    `UnsupportedOperationException` otherwise.

    :   **Returns:**: the exception that occurred during conversion **Throws:**: `java.lang.UnsupportedOperationException` - if the type of failure is not `SyncFailureType.CONVERSION_FAILED`

---


### getValidationResult

```
public Validator.Result getValidationResult()
```

:   Returns the result that was returned from the
    `Binding's` validator if this failure represents a
    validation failure. Throws `UnsupportedOperationException` otherwise.

    :   **Returns:**: the result that was returned from the `Binding's` validator **Throws:**: `java.lang.UnsupportedOperationException` - if the type of failure is not `SyncFailureType.VALIDATION_FAILED`

---


### toString

```
public java.lang.String toString()
```

:   Returns a string representation of the `SyncFailure`. This
    method is intended to be used for debugging purposes only, and
    the content and format of the returned string may vary between
    implementations. The returned string may be empty but may not
    be `null`.

    :   **Overrides:**: `toString` in class `java.lang.Object`
    :   **Returns:**: a string representation of this `SyncFailure`


---


|  |  |  |
| --- | --- | --- |
| **Overview** \| **Package** \| **Class** \| **Use** \| **Tree** \| **Deprecated** \| **Index** \| **Help** | |  |
| **PREV CLASS**   **NEXT CLASS** | **FRAMES**    **NO FRAMES**     **All Classes** |  |
| SUMMARY: NESTED \| FIELD \| CONSTR \| METHOD | DETAIL: FIELD \| CONSTR \| METHOD |  |


---
